# Race/Ethnicity and gender differences in health intentions and behaviors regarding exercise and diet for adults with type 2 diabetes: A cross-sectional analysis

**DOI:** 10.1186/1471-2458-11-533

**Published:** 2011-07-05

**Authors:** James R Gavin, Kathleen M Fox, Susan Grandy

**Affiliations:** 1 Emory University School of Medicine, 1648 Pierce Drive NE, Atlanta, GA 30322, USA; 2 Strategic Healthcare Solutions, LLC, PO Box 543, Monkton, MD 21111, USA; 3 AstraZeneca Pharmaceuticals LP, 1800 Concord Pike, Wilmington, DE 19850, USA

**Keywords:** type 2 diabetes, racial differences, exercise, weight management

## Abstract

**Background:**

Self-management is the cornerstone of diabetes control and prevention of complications; however, it is undetermined whether differences in intention to adopt healthy lifestyles and actual healthy behavior exist across race/ethnic groups. This study evaluated the differences across racial-ethnic groups in self-reported medical advice received and health intentions and behaviors among adults with type 2 diabetes mellitus.

**Methods:**

A cross-sectional analysis of the 2007 SHIELD US survey ascertained self-reported health intentions and behaviors for regular exercise, diet, and weight management among Non-Hispanic Caucasian (n = 2526), Non-Hispanic African-American (n = 706), and Hispanic (n = 179) respondents with type 2 diabetes.

**Results:**

A similar proportion of respondents from each race-gender group (43%-56%) reported receiving healthcare advice to increase their exercise (P = 0.32). Significantly more minorities reported an intention to follow the exercise recommendation compared with Non-Hispanic Caucasians (P = 0.03). More Non-Hispanic African-American (29%) and Hispanic (27%) men reported exercising regularly compared with other race-gender groups (P = 0.02). Significantly more Non-Hispanic Caucasian women (74%) and Hispanic women (79%) reported trying to lose weight compared with other groups (P < 0.0001).

**Conclusions:**

Differences in health intentions and healthy behaviors were noted across race-gender groups. More Non-Hispanic African-American men reported an intention to follow advice on exercising and self-report of exercising regularly was also higher compared with other race-gender groups. More Hispanic men reported high physical activity levels than other groups. Despite an increased willingness to follow healthcare recommendations for diet, >50% of respondents were obese among all race-gender groups.

## Background

In the United States, considerable variation by race and ethnicity exists in healthcare access and utilization for a number of diseases and conditions [1-4]. It is also well established that racial and ethnic differences exist in end-stage clinical outcomes for patients with diabetes. Microvascular complications of retinopathy, neuropathy, and nephropathy are more common in African-Americans, Hispanics, and Native Americans with diabetes than in non-Hispanic Caucasian patients [5-7]. Additionally, studies have found lower proportions of African-Americans and Mexican-Americans monitoring their blood glucose, having their cholesterol checked, or having their dyslipidemia diagnosed compared with Caucasians [4,8,9]. Exercise and physical activity are important for the management of type 2 diabetes mellitus and related complications, lowering the risk of mortality and incidence of diabetes [10-13]. Yet, only 39% of adults with diabetes engage in regular physical activity [14]. Approximately two-thirds of US adults with diabetes have body mass index of 27 or greater, indicating overweight or obese [15]. The current contribution of the healthcare system and physician-patient interaction to racial and ethnic differences in health outcomes such as exercise and obesity versus the contribution of patient self-care practices is yet undetermined.

To investigate whether there are differences across racial-ethnic groups in the self-reported information provided to adults with type 2 diabetes mellitus by their healthcare providers or differences in the health intentions and behaviors among these individuals, we analyzed data from the **S**tudy to **H**elp **I**mprove **E**arly evaluation and management of risk factors **L**eading to **D**iabetes (SHIELD). SHIELD, a large US population-based survey, provides longitudinal data on healthcare providers' health recommendations, individuals' intention to follow the health recommendations, and actual health behaviors practiced by adults with type 2 diabetes. It is undetermined whether physicians provide similar health recommendations to their patients with type 2 diabetes who are Caucasian versus minorities and whether minority patients intend to and actually follow the recommendations compared with Caucasians.

## Methods

A cross-sectional analysis of the 2007 SHIELD survey data was conducted to determine if differences exist across racial-ethnic groups for self-reported clinical advice from healthcare providers regarding diet and exercise and respondents' intention to follow the advice and their health behaviors among individuals with self-reported diagnosis of type 2 diabetes.

### SHIELD surveys

SHIELD included an initial screening phase to identify cases of interest in the general population (e.g., diabetes mellitus), a baseline survey to follow up identified cases with a questionnaire about health status, health knowledge and attitudes, and current behaviors and treatments, and annual follow-up surveys. A detailed description of the SHIELD methodology has been published previously [16,17].

In brief, the screening survey was mailed on April 1, 2004, to a stratified random sample of 200,000 U.S. households, representative of the U.S. population for geographic residence, household size and income, and age of head of household [18], identified by the Taylor Nelson Sofres National Family Opinion (TNS NFO) panel (Greenwich, CT). All TNS NFO surveys were voluntary, and no special incentives were provided. A response rate of 64% was obtained with 8.0% of the respondents having type 2 diabetes.

The baseline survey was mailed in September-October 2004 to a representative sample of individuals (n = 22,001) who were identified in the screening survey as having self-reported type 1 diabetes mellitus or type 2 diabetes mellitus, no diabetes, or being at risk for diabetes. The baseline survey and the subsequent annual follow-up surveys were sent to the individuals of interest and not the original head of the household. Each respondent group was balanced to be representative of that population for age, gender, geographic region, household size, and income for the U.S. population, and then a random sample from each group was selected and sent the baseline survey. A response rate of 72% was obtained.

Annual follow-up surveys were mailed to all individuals from the baseline survey who were still enrolled in the TNS NFO panel. To better understand the role that race and ethnicity may have on disease and lifestyle management, the mailing for the 2007 survey was increased with a targeted sample of minorities (African-Americans and Hispanics). Minority respondents from the 2004 screening survey who had not been selected for the baseline survey were invited to complete the 2007 survey. From the supplemental minority sample, 4,125 African-American and 1,674 Hispanic respondents completed the 2007 survey. This analysis used the combined survey responses from the core 2007 sample and the supplemental minority sample, with a total response rate of 69%. Only respondents with a diagnosis of type 2 diabetes (n = 706 African-American and 179 Hispanic) were included in the analyses. The SHIELD study was approved by the Quorum Review Board.

### Study Measures

Respondents were classified as having type 2 diabetes based upon their self-report of having been told by a doctor, nurse, or other healthcare professional that they had type 2 diabetes. Medical advice regarding exercise and diet, an individual's intention to follow the recommendations, and current exercise and weight management were assessed. For exercise, respondents were asked, "In the past 12 months, has a physician, nurse or other health professional recommended that you increase the amount that you exercise?" (yes/no) and "Do you intend to follow their recommendations?" (yes/no). To capture attitudes about the benefits of exercise, respondents were asked, "How likely do you think it is that increasing the amount of exercise you do will keep you healthy?" Response categories included very unlikely, unlikely, don't know, likely, and very likely. Current exercise behavior was captured by asking, "Which of the following statements best describes your current exercise routine?: 1) I currently do not exercise and I do not intend to start exercising in the next 6 months; 2) I currently do not exercise, but I am thinking about starting to exercise in the next 6 months; 3) I currently exercise some, but not regularly; 4) I currently exercise regularly, but have only begun doing so in the last 6 months; and 5) I currently exercise regularly and have done so for longer than 6 months." Additionally, the type and length of physical activity over the previous 7 days was assessed through the 7-item International Physical Activity Questionnaire (IPAQ) short form [19]. The IPAQ scores are categorized into 3 levels: 1) low or inactive, 2) moderate activity of at least 600 metabolic equivalent (MET)-minutes/week (3 or more days of vigorous activity of at least 20 minutes per day or 5 or more days of moderate activity or walking of at least 30 minutes per day), and 3) high activity of a minimum of 1500 MET-minutes/week (vigorous activity on at least 3 days or 7 days of any combination of walking, moderate or vigorous activities). A continuous IPAQ score expressed as MET-minutes/week was calculated as the MET level multiplied by minutes of activity times events per week according to the scoring protocol.

For diet and weight management, respondents were asked, "In the past 12 months, has a physician, nurse, or other health professional recommended that you change what you eat?" (yes/no) and "Do you intend to follow their recommendation?" (yes/no). Weight management behavior was reported as the response to "have you tried to lose weight during the last 12 months?" (yes/no). Respondents were also asked, "In the past 12 months, have you tried to change anything in your diet or your physical activities in any way to improve your health?"(yes/no). Body mass index (BMI) was calculated based on self-reported height and weight. BMI scores were categorized as normal weight (BMI < 25.0 kg/m^2^), overweight (BMI 25.0-29.9 kg/m^2^), and obese (BMI ≥ 30.0 kg/m^2^).

Race and ethnicity were self-reported as two separate questions: 1) "Are you of Spanish/Hispanic descent?" and 2) "Of what race do you consider yourself?" Individuals who reported that they were of Spanish/Hispanic descent were categorized as Hispanic regardless of the race reported. Respondents were categorized as "Caucasian, non-Hispanic" if they reported Caucasian race and no Hispanic descent. Likewise, respondents were categorized as "African-American, non-Hispanic" if they reported African-American race and no Hispanic descent. Asian or Pacific Islander, American Indian, Aleut Eskimo or other race respondents (n = 130) were excluded from the analysis due to small sample and heterogeneity of these groups. Annual household income was self-reported in categories of <$30,000, $30,000-$49,999, $50,000-$74,999, and ≥$75,000. Education levels were classified as high school degree or less, some college, college degree, and graduate work or degree.

### Statistical Analysis

Analyses were stratified by gender and household income to assess whether physician recommendations and actual behavior differed between men and women and across income levels. Education level was not used in the stratification because of the high correlation between income and education. Racial-ethnic groups were compared by chi-square test for proportions and ANOVA for means. Statistical significance was established *a priori *as a P value < 0.05.

## Results

A total of 3,604 respondents with type 2 diabetes completed the 2007 SHIELD surveys. Along with the 130 patients excluded for "other" race, an additional 63 respondents were excluded due to incomplete responses to the survey questions of interest. Among this final cohort (n = 3,411) of type 2 diabetes, there were 2,526 (74%) non-Hispanic Caucasian, and 706 (21%) non-Hispanic African-American, and 179 (5%) Hispanic respondents.

Comparison of sociodemographic characteristics across the 3 racial-ethnic groups indicated that there were significantly more African-American women (67%) compared with Caucasian (58%) and Hispanic (55%) women (P < 0.01). Hispanics were slightly younger (mean age of 59 years) than African-Americans (62 years) and Caucasians (63 years) (P < 0.05). There were also significantly more African-Americans with low household income (<$30,000/year, 46%) compared with Caucasians (39%) and Hispanics (41%) (P < 0.01). Education level was similar across the 3 groups (P > 0.05).

### Exercise recommendation and behaviors

Overall 47% of respondents reported that a healthcare professional recommended an increase in the amount of exercise. A similar proportion of respondents from each racial-ethnic and gender group reported that a healthcare professional had recommended that they increase the amount that they exercise (43%-56%) (P = 0.32) (Table 1). Among those who reported that a healthcare professional recommended exercise, 33%-46% of respondents indicated that they intended to follow the exercise recommendation most or all of the time. Concerning respondents' intention to follow exercise recommendations, significant differences were observed across the groups (P = 0.03). More minorities (46% African-American men, 42% African-American women, 38% Hispanic men, 44% Hispanic women) reported that they intended to follow the exercise recommendation than Caucasians (34% men, 32% women). Overall, 67% of respondents indicated that it is either "likely" or "very likely" that increasing the amount of exercise would keep them healthy. However, significant differences were observed across the racial-ethnic and gender groups; fewer Caucasian men (64%) and more Hispanic women (75%) stated that increasing the amount they exercise would keep them healthy (P = 0.008) (Table 1).

**Table 1:** Proportion of respondents with type 2 diabetes reporting exercise advice from healthcare professional, intention to follow the advice, attitude toward exercise, and exercise behavior by race-gender groups

Exercise	Non-Hispanic Caucasian men N = 1053	Non-Hispanic Caucasian women N = 1466	Non-Hispanic African American men N = 235	Non-Hispanic African American women N = 470	Hispanic men N = 81	Hispanic women N = 98
**Healthcare professional recommended increase in exercise, % (SE)**	46.4 (0.02)	47.5 (0.01)	43.0 (0.03)	46.8 (0.02)	51.9 (0.06)	56.1 (0.05)

**Intend to follow exercise recommendation**^**a**^, % (SE)*	34.6 (0.02)	32.9 (0.02)	45.5 (0.05)	41.9 (0.03)	38.1 (0.08)	44.4 (0.07)

Think increasing exercise will keep me healthy (likely or very likely), % (SE)**	63.9 (0.02)	66.0 (0.01)	69.0 (0.03)	72.3 (0.02)	72.8 (0.05)	75.3 (0.04)

Exercise regularly, % (SE)*	24.8 (0.01)	20.7 (0.01)	28.5 (0.03)	26.0 (0.02)	27.2 (0.05)	21.4 (0.04)

Physical activity (IPAQ), % (SE)**						

**High physical activity level**	17.1 (0.01)	10.2 (0.01)	13.6 (0.02)	12.4 (0.02)	23.5 (0.05)	19.4 (0.04)

**Minimally active**	25.7 (0.01)	19.3 (0.01)	24.3 (0.02)	19.4 (0.02)	18.5 (0.05)	17.3 (0.04)

**Inactive**	57.2 (0.01)	70.5 (0.01)	62.1 (0.02)	68.2 (0.02)	58.0 (0.05)	63.3 (0.04)

*p < 0.03 for comparison across race-gender groups

**p < 0.001 for comparison across race-gender groups

a: Intention to follow exercise recommendation was answered only by respondents who received the recommendation

IPAQ: International Physical Activity Questionnaire

Current exercise behaviors differed significantly across racial-ethnic and gender groups (P = 0.02) (Table 1). More African-American men (29%) and Hispanic men (27%) reported exercising regularly compared with other race-gender groups. However, the proportion of African-American men (14%) doing high physical activity as indicated by the IPAQ scores was low and similar to Caucasian women (10%) and African-American women (12%) (P < 0.0001) (Table 1). Hispanic men had the greatest proportion doing high physical activity (24%). Among men, African-Americans have the largest proportion who were inactive using IPAQ. For women, Hispanics had the lowest proportion who were inactive. On average, Hispanic men expended 2346 MET-minutes/week, compared with 1765 MET-minutes/week for Caucasian men, 1586 MET-minutes/week for African-American men, and 1050-1521 MET-minutes/week for women of each racial group (P < 0.0001).

Among the subset of respondents who reported receiving medical advice to exercise and that they intended to follow the exercise recommendation (16-24% of each race-gender group), Hispanic men had the lowest proportion (19%) and Caucasian women had the highest proportion (36%) who reported intent to follow the recommendation and exercising regularly. Among men, 35% of Caucasians, 22% of African-Americans, and 19% of Hispanics reported intent to follow the recommendation and exercising regularly. For women, 36% of Caucasians, 28% of African-Americans, and 21% of Hispanics reported intent to follow the recommendation and exercising regularly.

### Diet recommendation and weight management

From 40% to 57% of respondents indicated that a healthcare professional had recommended that they change what they eat (Table 2). A greater proportion of Hispanic men (57%) and women (56%) reported being told to change what they eat compared with Caucasians (40% men, 43% women) and African-Americans (44% men, 49% women) (P < 0.0001). Of those who reported receiving the diet recommendation, 48%-72% of respondents indicated that they intended to follow the diet recommendation most or all of the time. More African-American women (72%) and fewer Caucasian men (48%) reported that they intended to follow the diet recommendation compared with the other race-gender groups (P < 0.0001) (Table 2).

**Table 2:** Proportion of respondents with type 2 diabetes reporting diet advice from healthcare professional, intention to follow the recommendation, trying to lose weight, and obesity by race-gender groups

Diet/Obesity	Non-Hispanic Caucasian men N = 1053	Non-Hispanic Caucasian women N = 1466	Non-Hispanic African American men N = 235	Non-Hispanic African American women N = 470	Hispanic men N = 81	Hispanic women N = 98
Healthcare professional recommended change in what you eat, % (SE)*	40.0 (0.02)	42.7 (0.01)	43.6 (0.03)	48.9 (0.02)	56.8 (0.06)	56.1 (0.05)

**Intend to follow diet recommendation**^**a**^, % (SE)*	48.2 (0.02)	62.5 (0.02)	62.4 (0.05)	71.8 (0.03)	54.3 (0.07)	57.4 (0.07)

Tried to lose weight in past 12 months, % (SE)*	62.5 (0.02)	74.3 (0.01)	54.1 (0.03)	65.8 (0.02)	58.0 (0.06)	78.7 (0.04)

**Obese (body mass index ≥30 kg/m**^**2**^), % (SE)*	55.3 (0.02)	71.5 (0.01)	52.0 (0.03)	67.0 (0.02)	56.3 (0.06)	68.8 (0.05)

*p < 0.0001 for comparison across race-gender groups

a: Intention to follow diet recommendation was answered only by respondents who received the recommendation

More than 50% of each race-gender group reported trying to lose weight in the past 12 months (Table 2). Significantly more Caucasian women (74%) and Hispanic women (79%) reported trying to lose weight compared with the other race-gender groups (P < 0.0001). African-American men (54%) and Hispanic men (58%) had the lowest proportions trying to lose weight. Actual BMI scores showed that more than 50% of all respondents were obese, with more women from each race being obese (P < 0.0001) (Table 2).

Health behavior for diet or physical activity varied across race-gender groups. Significantly more African-American women (78%) and fewer Hispanic men (49%) reported that they had tried to change their diet or physical activities to improve their health compared with the other race-gender groups (65% Caucasian men, 73% Caucasian women, 58% African-American men, 72% Hispanic women) (P < 0.0001). For the subset of respondents who reported receiving medical advice to change their diet and reported that they intended to follow the diet recommendation (19-35% of each race-gender group), African-American men had the lowest proportion (73%) and Hispanic women had the highest proportion (87%) who reported intent to follow the recommendation and tried to lose weight. Among men, 82% of Caucasians, 73% of African-Americans, and 76% of Hispanics reported intent to follow the recommendation and tried to lose weight. For women, 86% of Caucasians, 80% of African-Americans, and 87% of Hispanics reported intent to follow the recommendation and tried to lose weight.

Stratification by household income (<$30,000 and ≥$30,000) did not change the associations observed in the race-gender groups. The associations that were similar (P > 0.05) across race-gender groups (e.g., healthcare professional exercise recommendation) were similar for both income levels, and associations that were significantly different across race-gender groups (e.g., intention to follow recommendation, exercise behavior) remained significant for both income levels. Multivariable logistic regression model showed that after controlling for age, education, household income, and obesity, African Americans were more likely to report exercising regularly than Caucasian men but the association was only significant for African American women (odds ratio = 1.30, Table 3). After controlling for age, education, and household income, Caucasian and African American women were more likely to be obese than Caucasian men but the association was significant only for Caucasian women (odds ratio = 1.77, Table 3). African American men were less likely to be obese compared with Caucasian men (odds ratio = 0.72, Table 3).

**Table 3:** Multivariable logistic regression odds ratios for exercise regularly and for obesity, adjusting for age, education, and income

	Exercise regularly*		Obese	
**Race-gender group (reference group = Non-Hispanic Caucasian men)**	**Odds ratio (95% CI)**	**P value**	**Odds ratio (95% CI)**	**P value**

**Non-Hispanic Caucasian women**	1.02 (0.84-1.25)	0.82	1.77 (1.48-2.12)	<0.0001

**Non-Hispanic African American men**	1.25 (0.90-1.74)	0.18	0.72 (0.54-0.97)	0.03

**Non-Hispanic African American women**	1.30 (1.00-1.69)	0.05	1.25 (0.98-1.59)	0.07

**Hispanic men**	1.13 (0.67-1.91)	0.64	0.95 (0.59-1.54)	0.84

**Hispanic women**	1.13 (0.68-1.90)	0.63	1.07 (0.67-1.70)	0.78

*also adjusted for obesity

## Discussion

This study provides new evidence of racial and ethnic differences in health intentions and behaviors for adults with type 2 diabetes. Although there was no difference in the proportions reporting that a healthcare professional recommended increasing exercise across race-gender groups, more African-American men reported an intention to follow the exercise recommendation and a greater proportion reported that they currently were exercising regularly than other race-gender groups. More Hispanic men reported exercising regularly with high physical activity levels than other race-gender groups. Racial/ethnic differences were also evident for diet and weight management. More Hispanic men and women reported that a healthcare professional recommended changes in their diets despite similar BMI levels across groups. While more Caucasian women and Hispanic women reported trying to lose weight, African-American men and Hispanic men had the lowest proportion trying to lose weight. Household income did not impact these racial differences.

This study also highlights the discordance between individuals' impressions of what they are doing regarding healthy behaviors and their actual performance, a disconnect that appears to be more prominent among African-Americans. For example, more African-American men indicated that they were currently exercising regularly than other race-gender groups, but they had the lowest proportion of men with high physical activity levels and the lowest mean IPAQ scores among the men. In contrast, Hispanic men constituted a larger proportion of those who reported that increasing exercise will keep them healthy. They also reported exercising regularly and had the largest proportion with high physical activity.

A number of differences in intentions and approaches to weight loss were seen among the various groups. More Caucasian women and Hispanic women reported trying to lose weight, yet these race-gender groups had the highest proportion of respondents with obesity, indicating that intent did not translate to weight loss. In contrast, African-American men and Hispanic men reported the lowest proportion trying to lose weight. Although there were similar intentions regarding weight loss between the latter two groups, Hispanic men had the highest proportion of physical activity, whereas African-American men had lower proportions of high physical activity levels similar to those of Caucasian and African-American women. It is possible that the choice to pursue a high level of physical activity among Hispanic men is not connected to the desire for weight loss, but driven by other considerations.

There appears to be a willingness among some minority respondents to follow the recommendations of their healthcare providers regarding healthier lifestyle choices. Indeed, African-American women indicated greater intensions to change their existing diets and indicated that they currently exercise more than other groups. This willingness and intention to engage in healthy behaviors needs to be put into action given the health benefits of physical activity, exercise, and weight management for the management of type 2 diabetes [20]. There has been increased public health attention on the need to reduce obesity and increase physical activity because of the epidemic in diabetes of recent years [21]. Until intentions are translated to positive behaviors, there will be little improvement in the twin epidemics of diabetes and obesity. It is hypothesized that since advice is given by the healthcare professionals seen by the SHIELD respondents, there is a need to implement intentions to achieve behaviors. The lack of greater achievement of lifestyle behaviors suggests a need for more access to and use of appropriately designed tools for weight loss (e.g. prescribed diet plans) and exercise (e.g. exercise equipment, aerobic exercise videos), and fitness facilities or exercise programs at community centers or school gymnasiums for effective lifestyle modification among minority individuals. Several studies have indicated that tools and/or alternative methods to education beyond print materials have prompted behavior change. Tailored computer programs for setting goals for nutrition and physical activity which are reviewed at each visit by physicians resulted in increased physical activity and weight loss compared with printed health education materials among overweight patients with T2DM [22]. Step pedometers with and without print materials were effective for increasing physical activity compared with a standard public health recommendation for physical activity or print materials only [23]. Telemedicine, video education and daily text messages via mobile phones have prompted behavior adherence and weight loss compared with print materials and standard education [24-26]. Among stroke patients, an education session did not result in positive diet or physical activity changes [27]. Other studies indicate that education sessions and materials need to be tailored, culturally appropriate with personally relevant information for work and home lives to improve motivation and health behavior change [28-30].

Data from the 2002 Medical Expenditure Panel Survey (MEPS) indicated that 73% of adults with diabetes were told by a health professional to exercise more [14] which is higher than the approximate 50% of respondents in the present study. Additionally, the MEPS study indicated that the likelihood of receiving medical advice on exercise was less likely in Hispanics but the present study found a similar percentage receiving advice across the race-gender groups. The differences may be due to more current data (2007) in SHIELD and differences in population composition (e.g. age, gender, race). The present study found that <30% of respondents reported exercising regularly and <20% reported high physical activity compared with 39% of adults with diabetes reporting being physically active in the MEPS 2003 survey [31]. The 2000 Behavioral Risk Factor Surveillance System (BRFSS) national survey reported that about 30% of the US adults exercised regularly and 14% did intense physical activity [21]. The MEPS study [31] indicated that Hispanics with diabetes were potentially more likely than whites (odds ratio = 1.43, 95% CI: 0.98-2.07) to be physically active while the SHIELD study found a greater percentage of Hispanics, especially men, had high physical activity compared with other race groups. For weight control and diet, the BRFSS reported that 38% of US adults were trying to lose weight, which increased to 66% of obese adults, compared with 60-70% of SHIELD T2DM (mostly obese) respondents reporting trying to lose weight. Overall, the SHIELD findings are similar to other national surveys but differences may be due to change over time (2000-2003 to 2007) and different exercise/physical activity questions being utilized in the different surveys.

The present study has limitations that should be considered. The determination of type 2 diabetes and obesity were made based upon self-report rather than clinical or laboratory measures; therefore, there may be misclassification bias. However, all respondents were asked the same questions to assess diabetes and weight status. Medical advice, physical activity, diet, and weight management were also self-reported. However, the IPAQ for physical activity assessment is a validated and well-accepted physical activity instrument. Household panels such as the SHIELD study tend to under-represent the very wealthy and very poor segments of the US population and do not include military or institutionalized individuals. Thus, the study findings should not be generalized to these population segments. The survey was provided in English only, thus potentially excluding individuals who spoke other languages, especially among Hispanic households. Given that the supplemental minority sample that was added in 2007 was not part of the random, stratified sampling method at baseline, these minority respondents may not be representative of the African-American or Hispanic populations in the United States. This study examined health intentions and behaviors by race-gender groups which may not represent individual intent-behavior correlation.

## Conclusions

In conclusion, differences in health intentions and actual healthy behaviors were noted across racial and gender groups. More Non-Hispanic African-American men reported an intention to follow advice on exercising and they reported a greater frequency of exercising than other race-gender groups. More Hispanic men reported high physical activity levels than other groups. Despite an increased willingness to follow healthcare recommendations for diet, more than 50% of respondents were obese among all race-gender groups. More research is needed to better understand how to assist communities in translating intentions for lifestyle change into achievement of healthy behaviors.

## Competing interests

Dr. Gavin is an advisor to AstraZeneca LP. Dr. Fox received research funds from AstraZeneca LP to conduct the analysis. Dr. Grandy is an employee and stockholder of AstraZeneca LP.

## Authors' contributions

JRG participated in the conception and design of the SHIELD study, assisted in the interpretation of the study data, and critically reviewed and revised the manuscript. KMF participated in the design of the investigation, performed the statistical analysis and data interpretation, and drafted the manuscript. SG conceived of the SHIELD study, participated in its design and coordinated the acquisition of data, and critically reviewed and revised the manuscript. All authors read and approved the final manuscript.

## Pre-publication history

The pre-publication history for this paper can be accessed here:

http://www.biomedcentral.com/1471-2458/11/533/prepub
